# Urinary bisphenol levels and blood pressure after soda consumption from cans, PET and glass bottles

**DOI:** 10.1038/s41598-025-18459-z

**Published:** 2025-09-12

**Authors:** Leonie Plachetka, Virginie Stanislas, Alexander Bauer, Thomas Göen, Heike Denghel, Karin B. Michels

**Affiliations:** 1 Institute for Prevention and Cancer Epidemiology, Medical Center – University of Freiburg, Faculty of Medicine, University of Freiburg, Elsaesserstr. 2, 79110 Freiburg, Germany; 2 Institute and Outpatient Clinic of Occupational, Social and Environmental Medicine, Friedrich-Alexander-Universität Erlangen-Nürnberg, 91054 Erlangen, Germany

**Keywords:** Risk factors, Diseases, Cardiovascular diseases, Endocrine system and metabolic diseases

## Abstract

Bisphenol A (BPA) is an endocrine-disrupting chemical (EDC) that has been associated with various human disorders. Human exposure is ubiquitous. The primary route of exposure is ingestion, as BPA can leach from plastic food packaging and the epoxy resin lining of cans into food. Restrictions on the use of BPA in consumer products due to its health concerns have resulted in the development and introduction of new BPA alternatives. These compounds are likely to have health effects comparable to those of BPA. However, data on the type and amount of substitutes used in consumer products are difficult to obtain, and studies on their prevalence of use and impact on health in human populations are sparse. The aim of the present study was to evaluate the urinary concentrations of BPA and its analogues after the consumption of soda from cans compared to polyethylene terephthalate (PET) bottles and glass bottles. Additionally, we investigated blood pressure levels before and after soda consumption from cans, PET bottles, and glass bottles. We conducted a randomized crossover trial with 103 female participants between 30 and 65 years of age. The participants were assigned to drink soda (Coca-Cola light) from cans, PET bottles and glass bottles on two days of an intervention week. Urine samples were collected two to three hours after the intervention. Blood pressure levels were measured before and two to three hours after the intervention. Urinary bisphenol concentrations were measured via GC-AEI-MS/MS. The associations between the intervention and log10-transformed bisphenol concentrations and blood pressure levels were evaluated via a mixed models approach. For comparisons of the interventions, we used Tukey tests. We found significantly higher BPA concentrations in the urine samples of our study participants after soda consumption from cans than after soda consumption from PET bottles (+ 22.3%, 95% CI 3.37;44.64, P value 0.014). There was no difference compared to glass bottles. In addition to BPA, we detected BPF, BPS and BPE in some of the urine samples, but to a lesser extent, and independent of beverage packaging. Systolic and diastolic blood pressure increased across all interventions independent of whether soda was consumed from cans, PET bottles, or glass bottles. This study suggests that canned soda may increase the amount of BPA excreted in the urine of humans. The increase in blood pressure, independent of beverage packaging, may be due to the caffeine content of the consumed beverage. Given the potential of bisphenol exposure from canned food and beverage consumption, as well as the availability of alternatives in the epoxy resin lining of cans, complete removal of BPA from cans is an attractive option for mitigating human exposure to bisphenols.

Universal Trial Number/ DRKS-ID: U1111-1244-7033/ DRKS00019922 (Registration Date 29/11/2019).

**Supplementary Information:**

The online version contains supplementary material available at 10.1038/s41598-025-18459-z.

## Introduction

Bisphenols are a group of monomers with similar structures of two phenolic rings connected by a variable chemical group. The most common and most studied member of this group, bisphenol A (BPA), is obtained by the condensation of two phenolic rings and an acetone molecule^1^. This structure gives BPA the ability to mimic estrogen activity and act as an endocrine disrupting chemical (EDC)^2,3^. Through binding to estrogen receptors, it is thought to disrupt normal cell function by signaling estrogen activity and regulating gene expression. Simultaneously, BPA competes with estrogen for the binding of membrane estrogen receptors and therefore blocks estrogen activity. Furthermore, it acts as an androgen antagonist and is assumed to interact with a range of other cellular processes^4–7^.

Epidemiologic studies suggest associations of BPA exposure with various diseases, such as hormone-sensitive cancers, reproductive disorders and metabolic diseases, such as diabetes, obesity, and cardiovascular diseases^8–12^. Hypertension is one of many risk factors for cardiovascular disease. A positive association between urinary BPA levels and hypertension, independent of traditional risk factors, was first suggested by Shankar and Teppala in 2012 and subsequently reported by others^9,13,14^. However, a study using other criteria and definitions did not support these findings^15^. Moreover, various possible molecular mechanisms may explain any effect of BPA on blood pressure and cardiovascular disease, but this is not fully understood^16,17^. As the prevalence and health burden of these diseases are increasing, further understanding of the impact of bisphenols on human health is sensible^18^.

The primary human exposure route for BPA is ingestion, followed by dermal contact and inhalation^19–21^. It is a high production chemical with an estimated compound annual growth rate of at least 3.5% in the forecast period until 2032^22^. BPA is used as a monomer in the manufacture of polycarbonate plastic and epoxy resins^23,24^, which are used in a variety of products, including dental composites and sealants and food contact materials such as the inner lining of cans or plastic food packaging^25^. Until January 2020, BPA was also used as a color-developing agent in thermal paper, but its use was restricted in some countries to not contain more than ≥ 0.02% by weight due to health concerns^26^.

Incomplete polymerization or factors such as salinity, acidity and the presence of vegetable oil in canned goods can result in the leaching of BPA into food and beverages^27–29^. Previous crossover intervention trials reported significant increases in urinary BPA concentrations following the consumption of canned products compared to fresh alternatives and products in glass packaging^9,30^. Furthermore, one of these studies found that BPA uptake was associated with elevated systolic blood pressure levels^9^.

BPA is rapidly metabolized by glucuronidation after oral uptake, its biological half-life is approximately 6 h, and it is nearly completely eliminated within 24 hours^31^. Nevertheless, various observational studies detected urinary BPA concentrations in more than 90% of European, North American, and Asian populations^32,33^, suggesting continuous and widespread exposure.

Owing to its potential adverse health effects, the use of BPA in consumer products has been restricted. The European Chemical Agency (ECHA) listed BPA on the candidate list of substances of very high concern in 2017^34^. Due to studies showing harmful effects of low doses of BPA on the immune system, the European Food Safety Authority (EFSA) lowered the tolerable daily intake of BPA from 4 µg/kg body weight to 0.2 ng/kg body weight in 2023^35–37^. However, low-dose effects of EDCs have been reported, and hormone-like chemicals display nonmonotonic dose‒response curves, compromising the ability to define a cutoff level at which these chemicals may be safe for human health^3^. In addition, the effects induced by BPA can appear years after exposure and can differ depending on different life stages. Children and women of reproductive age are likely particularly susceptible^32,38^.

Conversely, the restrictions in the use of BPA in consumer products have resulted in the development and introduction of new BPA alternatives on the market. Most of these include only few structural changes, and the main alternatives are bisphenols F (BPF) and S (BPS)^39,40^. An increase in the use of these agents has been observed^41^. Unfortunately, these substitutes are likely to have health effects comparable to those of BPA^42^. As data on the type and amount of substitutes used in consumer products are difficult to obtain, studies on their prevalence of use and impact on health in human populations are sparse.

We conducted a randomized crossover intervention trial to evaluate the urinary concentrations of BPA and its analogues after the consumption of soda from cans, PET bottles, and glass bottles. As a secondary objective, we investigated blood pressure levels before and after soda consumption from cans, PET bottles, and glass bottles.

## Materials and methods

### Study participants

**Fig. 1:**
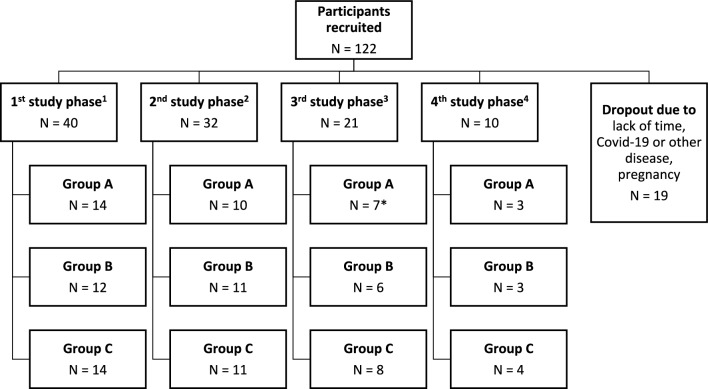
Flow chart illustrating the inclusion of study participants and their assignment to study phases and groups. ^1^ January and February 2020, ^2^ June and July 2020, ^3^ October 2020 until May 2021 (with a COVID-19 break in between the study period), ^4^ June and July 2021. *One participant had missing urine samples and was removed from the bisphenol analysis.

Female volunteers between 30 and 65 years of age were recruited between November 2019 and April 2021 via advertisements in local newspapers, flyers in public places, social media platforms, and the intranet of the university hospital in Freiburg, Germany. Volunteers with a systolic blood pressure ≥ 129 mmHg, cancer, diabetes, gastrointestinal disease, heart disease or kidney disease were excluded from the study. Additional exclusion criteria were pregnancy, smoking, and the intake of blood pressure-lowering medication or hormone replacement therapy during menopause. Menopausal status was assessed by the self-reported absence of menstrual bleeding for more than 12 months. For logistic reasons and owing to safety measures pertaining to the COVID-19 pandemic, we split the study into four study phases, with volunteers participating in the study either in 1) January and February 2020, 2) June and July 2020, 3) October 2020 until May 2021 (with a COVID-19 break in between the study period) or 4) June and July 2021. Depending on the time point at which the participants were recruited, they took part in study phases 1 to 4. Within the study phases, the study coordinator randomly assigned each participant a number from 1 to 3 consecutively as they were included in the study by the study team. This determined the group (A-C) to which they were assigned (Fig. 1). Additional information about the study participants, drop outs and power calculations is provided in the supplementary materials (Supplementary Material, Table S2).

### Intervention

For the intervention, we selected Coca-Cola light. Germany is one of the top countries for soda sales in Europe, with Coca-Cola being the most commonly used soft drink brand worldwide in 2020^43,44^. Coca-Cola light is available in cans, PET bottles, and glass bottles. We purchased the entire quantity needed for the study at one timepoint to exclude any batch effects due to any change in the manufacturing process. Coca-Cola light has a pH value of approximately 3^45^.

### Study design

**Fig. 2:**
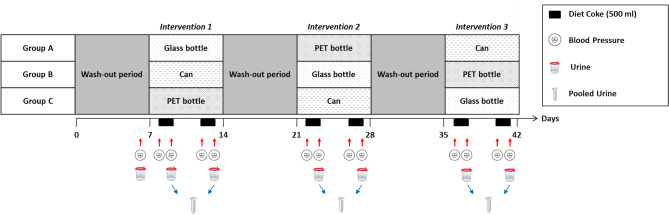
Study design of the randomized, single-blind, crossover intervention trial. Initially, all the participants in groups A-C (group A: top row; group B: middle row; and group C: bottom row) underwent a one-week wash-out period. At the end of this week, blood pressure was measured, and urine samples were taken. Group A began the intervention with Coca-Cola light from glass bottles, followed by Coca-Cola light from PET bottles and, finally, Coca-Cola light from cans on two days in each of the intervention weeks. Blood pressure was always measured shortly before consumption of Coca-Cola light and two to three hours after consumption. Urine samples were collected two to three hours after each intervention. The urine samples from the two intervention days per intervention week were combined. There was a one-week wash-out period between each intervention week. The same study protocol was used for groups B and C; only the order of the interventions differed from that of group A because of the crossover design. PET- Polyethylene terephthalate.

The study was conducted at the Institute for Prevention and Cancer Epidemiology, Faculty of Medicine and Medical Center, University of Freiburg in Freiburg, Germany. We used a randomized, single-blind, crossover intervention trial design (Fig. 2).

The study protocol was approved by the Ethics Committee of the University Hospital Freiburg. All experiments were conducted in accordance with the principles outlined in the Declaration of Helsinki and in compliance with Good Clinical Practice guidelines. The study was registered 29/11/2019 (DRKS00019922, UTN U1111-1244-7033). Informed consent was obtained from all study participants.

After a one-week wash-out period, the study participants were randomly assigned to one of three intervention groups that differed in the sequence of the three interventions they received. Starting with the first wash-out period and throughout the entire duration of the study, the participants were asked not to consume food or drinks from cans and avoid touching thermal receipt paper. The participants kept a food diary on the days before the urine collection.

At the end of the first wash-out period, prior to the start of the first intervention week, one initial urine sample was collected from each participant. Additionally, weight, height and blood pressure were assessed. Owing to the COVID-19 break in the 3rd study phase (October 2020 to May 2021), we collected an additional urine sample and measured the blood pressure of each participant after the first wash-out period after the resumption of the study (Supplementary Material, Fig. S1).

For the first intervention week of the study, one group (group A) was assigned to consume 500 mL of Coca-Cola light from glass bottles on two days of the first intervention week (either Monday and Thursday or Tuesday and Friday), with two days in between. The second group (group B) was assigned to consume 500 mL of Coca-Cola light from cans, and the third group (group C) was assigned to consume 500 mL of Coca-Cola light from PET bottles. We monitored adherence to the intervention as participants consumed the Coca-Cola light at our study center. The participants were blinded to the packaging type of the Coca-Cola light by transferring the beverages from the original packaging into regular drinking glasses before giving them to the participants for consumption. The participants were asked not to eat or drink any other than water between the intervention and the urine sample collection. There was a wash-out period of one week between each intervention week. During the second and the third intervention week of the study, the assignments were reversed (Fig. 2).

Urine samples from the participants were collected two to three hours after the intervention on both intervention days of each intervention week and stored in polypropylene cups. The urine sample from the first day of each intervention week was stored at 4 °C immediately after collection until the urine sample from the second day of the intervention week was collected. Both urine samples were subsequently pooled with equal shares of each sample to minimize within-person variation. The samples were stored in aliquots at −80 °C until further analysis.

### Blood pressure measurement

Prior to each intervention and after urine collection two to three hours post intervention, blood pressure was assessed using an automated sphygmomanometer (Medicus X, Bosch + Sohn GmbH) with a standard cuff. The participants had to rest for 5 min until the first measurement. Blood pressure was always measured at the left arm, and the participants were seated in a comfortable position. Overall, three blood pressure measurements were taken at each point in time, with one minute between each measurement.

### Analysis of urine samples

Bisphenol concentrations were assessed in urine samples at the Institute and Outpatient Clinic of Occupational, Social and Environmental Medicine, Friedrich-Alexander-Universität Erlangen-Nürnberg, 91,054 Erlangen, Germany, using a modification of a previously published method^46^. The concentrations of the bisphenols A, F, AF, S, E, C, B, P, Z, AP and BP were assessed. The following description focuses on the analysis of BPA and BPF as the other types of investigated chemicals (bisphenols AF, S, E, C, B, P, Z, AP and BP) presented high rates of undetected concentrations (> 90%) and were not considered for further analysis. Additional information on the measurement of these compounds can be found in the supplementary material.

#### Standards, reagents and analytical equipment

Standards for Bisphenol A (BPA, 2,2-bis(4-hydroxyphenyl)propane, purity 99%), and Bisphenol F (BPF, 4,4′-methylendiphenol, purity 98%), were purchased from Sigma-Aldrich (Steinheim, Germany).

Matched isotope-labeled standards of Bisphenol F—^13^C_12_ (BPF—^13^C_12_, chemical purity > 98%, isotopic purity 99%) and 3,3,5,5-Tetrabromobisphenol A—^13^C_12_ (TBBA—^13^C_12_, chemical purity > 98%, isotopic purity 99%) were obtained from Cambridge Isotope Laboratories (Andova, USA). Bisphenol A—^13^C_12_ (BPA—^13^C_12_, chemical purity 98%, isotopic purity 98.5%) was purchased from Biozol (Eching, Germany).

N-tert-Butyldimethylsilyl-N-methyltrifluoracetamid with 1% tert-butyldimethylchlorsilan (MtBSTFA + 1% TBDMSCl) as well as N,O-bis-(trimethylsilyl)-trifluoracetamide (BSTFA) were acquired for derivatization from Sigma‒Aldrich (Steinheim, Germany). For hydrolysis, β-glucuronidase/arylsulfatase from *Helix pomatia* was obtained from Roche Diagnostics GmbH (Mannheim, Germany).

Acetonitrile (ACN, anhydrous, GC grade), acetic acid (glacial), methanol (MeOH, anhydrous, GC grade) and toluene (anhydrous, GC grade) as well as sodium acetate (NaAc, for analysis) were purchased from Merck KGaA (Darmstadt, Germany). High-purity water was obtained from VWR (Darmstadt, Germany). Polystyrene divinylbenzene copolymer columns for solid-phase extraction (SPE) (Isolute® 101, 100 mg sorbent, 1 mL capacity, average particle size 65 µm (irregularly shaped particles), pore diameter 100 Å) were supplied by Biotage AB (Uppsala, Sweden).

Gas chromatogram-tandem mass spectrometry (GC-MS/MS) analysis was carried out using a Thermo Scientific TRACE 1310 gas chromatograph equipped with a split/splitless injector with a deactivated single taper helix liner, a Thermo Scientific TriPlus RSH autosampler and Chromeleon Software Version 7.2.8 for device control and data analysis and a Thermo Scientific TSQ 9000 triple quadrupole mass spectrometer with an advanced electron ionization (AEI) source installed (Thermo Fisher Scientific Waltham, USA). BPA levels were verified using an additional GC-MS/MS procedure^47^.

#### Sample preparation

Glassware was heated to 150 °C over night before use. Equipment for the solid phase extraction was rinsed with water, MeOH and acetone before use and was used exclusively for the presented method. Consumables, such as pipet tips, SPE cartridges, vials and screw caps were stored separately from other materials in the laboratory to avoid cross contamination. Solvent bottles were labeled and used only for this analysis procedure. Hydrolysis buffer and further aqueous solutions were prepared with high-purity water. Furthermore, only disposable nitrile gloves were worn during laboratory work.

For every sample preparation series, a reagent blank was included to assess possible sample contamination with ubiquitous free BPA or its surrogate substances. The concentrations of the analytes in the reagent blank were determined using the calibration curve and subsequently subtracted from the analyte concentrations in the urine samples to calculate the actual sample concentrations.

Frozen urine samples were thawed, equilibrated to room temperature and mixed by vortexing. This was followed by hydrolysis of phase II conjugates: 10 µL of internal standard working solution, 500 µL of 0.4 M NaAc buffer (pH 5) and 10 µL of β-glucuronidase/arylsulfatase from *Helix pomatia* were added to aliquots of 1 mL urine in 1.8 mL screw glass vials and samples were then incubated over night at 37 °C.

#### SPE, derivatization and GC-AEI-MS/MS analysis

For SPE, Isolute 101 cartridges were preconditioned with 500 µL MeOH, 250 µL ACN and twice with 500 µL of 0.4 M NaAc buffer (pH 5) and subsequently loaded with the hydrolyzed urine samples. Cartridges were then washed with 500 µL buffer, 750 µL double-distilled water and 500 µL 50% MeOH. Afterwards, the SPE cartridges were centrifuged for 10 min at 1900 × g and dried for 10 min under vacuum. Samples were eluted with 4 × 200 µL of ACN into a 1.8 mL screw glass vial containing 200 µL of toluene as a keeper. Eluates were concentrated to a volume of 100 µL at room temperature under a gentle stream of nitrogen. Derivatization was performed by adding 30 µL BSTFA to the concentrated sample extracts. After gentle vortexing and a reaction time of 10 min at room temperature, the samples were transferred into micro inserts and measured via GC-AEI-MS/MS.

#### Data preparation of urinary bisphenol concentrations

For both BPA and BPF values below the limit of quantification (LOQ), we imputed a value of the LOQ divided by the square root of 2 to undetected bisphenol concentrations (BPA: n = 232 urine samples (56.9%), BPF: n = 307 urine samples (75.3%); LOQ_BPA:_ 0.34 μg/L, LOQ_BPF:_ 0.22 μg/L.)^48^. The imputed bisphenol concentrations were subsequently adjusted for creatinine concentrations (bisphenol concentration in mpg (µg/L) divided by the creatinine concentration (g/L)).

### Statistical analysis

All statistical analyses were performed in R (version 4.1.1). Demographic data are presented as the median (1st quartile, 3rd quartile) for continuous variables, and as absolute frequencies and percentages for categorical variables. Data estimated using mixed effects models are presented as marginal means and standard error.

Differences between the study groups at baseline were evaluated by Kruskal–Wallis rank sum test for continuous variables and Fisher’s exact test for categorical variables.

Log10-transformed bisphenol concentrations were used as outcomes in the statistical models. The associations between the interventions and log10-transformed bisphenol concentrations were evaluated through the use of mixed models (R package lme4). Period, intervention and initial baseline (log10-transformed) were included as fixed effects. Plausible covariates were considered as potential additional fixed effects (Age, BMI, waist circumference and menopause) but were not retained in our model, as they did not enhance model fit. Interaction effects with these covariates were not explored due to the high amount of missing values, which could complicate model interpretation. Repeated measurements of each participant were taken into account by a random intercept term in all models. Global P values for fixed effects from the final models were obtained via analysis of variance (type III Wald’s χ2 test) from the car package. We estimated marginal means for each level of the dependent variable intervention and conducted pairwise comparisons using Tukey tests (function pairs.emmGrid from R package emmeans). We evaluated the reproducibility of our bisphenol measurements by measuring all urine samples from 12 randomly selected participants in duplicate and using Pearson correlation coefficients and intraclass correlation coefficients (ICCs) for comparison. ICCs were calculated based on a two-way random effects model with multiple raters and absolute agreement (R package irr). Bisphenol values were log10-transformed to address non-normal distribution. To investigate the potential impact of splitting the study population into four different study phases, the final model was re-estimated including an additional interaction effect between the intervention and the study phase. This new model was then compared to the original one using a likelihood ratio test.

For blood pressure analysis, we used the means of the last two systolic and diastolic blood pressure measurements. The means obtained over the two days of each intervention period were subsequently averaged to generate a representative mean blood pressure value before and after the intervention. We defined ΔBP as the difference between blood pressure after and blood pressure before intervention and used it as an additional outcome in our analysis. We conducted the same assessment for blood pressure changes as for changes in bisphenols. Fluctuations in blood pressure within and between participants over time were evaluated graphically (figure not shown) and did not show any irregularities.

The association between intervention and blood pressure (either blood pressure after intervention or ΔBP) was evaluated through similar mixed models incorporating period, intervention and initial baseline as fixed effects along with a random intercept term. Plausible covariates were considered as fixed effects and BMI was subsequently included as a fixed effect when blood pressure after intervention was set as the outcome as it improved model fit. Interaction effects were not explored. Global P values were obtained via ANOVA, and marginal means for each level of the intervention variable were estimated and compared via Tukey tests.

## Results

In total, 103 women with a median age of 46 years completed the study. The basic characteristics of the study participants at baseline are presented in Table 1. No significant difference were detected between the three study groups (P values not shown). BPA was detected in 43.1% and BPF in 24.8% of all the urine samples. Additionally, we detected BPS in 10.3% of all urine samples and BPE in 0.47% of all urine samples. Pearson correlation coefficients between duplicated urinary samples from 12 randomly selected participants were 0.98 for BPA, 0.99 for BPF, and 0.99 for creatinine. The corresponding ICCs (ICC values and confidence intervals) calculated on the same duplicated data, were 0.992 [0.979, 0.997] for BPA, 0.998 [0.992, 0.999] for BPF, and 0.990 [0.981, 0.994] for creatinine, indicating excellent reproducibility of the measurement methods. All data shown are urinary bisphenol concentrations imputed and normalized to creatinine. The median baseline (before intervention) urinary concentration of BPA was substantially higher than that of BPF [0.90 µg/g creatinine vs. 0.48 µg/g creatinine].

**Table 1:** Population characteristics at baseline.

Characteristic	Total study population, N = 103	Study group A, N = 34	Study group B, N = 32	Study group C, N = 37
Age^1^	46 (36, 54)	45 (35, 54)	46 (37, 54)	48 (37, 54)
BMI^1^	23.41 (21.73, 25.46)	23.00 (21.70, 25.02)	23.38 (21.81, 25.70)	23.73 (21.91, 25.80)
Menopause^2^				
Surgical	4 (3.9%)	1 (2.9%)	0 (0%)	3 (8.1%)
Post	31 (30%)	11 (32%)	9 (28%)	11 (30%)
Pre	68 (66%)	22 (65%)	23 (72%)	23 (62%)
BPA (normalized, [µg/g creatinine])^1^	0.90 (0.64, 1.27)	1.06 (0.50, 2.32)	0.84 (0.61, 1.00)	1.14 (0.71, 2.01)
< LOQ (0.34 μg/L)^2^	49 (48%)	20 (59%)	11 (34%)	18 (49%)
BPA (imputed and normalized, [µg/g creatinine])^1^	0.90 (0.63, 1.34)	1.09 (0.56, 1.50)	0.83 (0.60, 1.04)	1.03 (0.63, 1.46)
Unknown	1	1	0	0
BPF (normalized, [µg/g creatinine])^1^	0.63 (0.29, 1.86)	1.32 (0.40, 6.35)	0.54 (0.34, 0.92)	0.44 (0.22, 0.68)
< LOQ (0.22 μg/L)^2^	72 (70%)	22 (65%)	20 (63%)	30 (81%)
BPF (imputed and normalized, [µg/g creatinine])^1^	0.48 (0.26, 0.85)	0.71 (0.32, 1.04)	0.44 (0.22, 0.70)	0.41 (0.29, 0.68)
Unknown	1	1	0	0
Systolic blood pressure [mmHg]^1^	118 (113, 124)	120 (115, 124)	118 (111, 123)	118 (112, 122)
Diastolic blood pressure [mmHg]^1^	80 (74, 85)	81 (75, 86)	80 (74, 85)	80 (74, 85)

^1^ Median (1st quartile, 3rd quartile).

^2^ n (%).

Intervention scheme: Group A, glass-PET-can; Group B, can-glass-PET; Group C, PET-can-glass.

*BMI* body mass index, *LOQ* limit of quantification, *BPA* bisphenol A, *BPF* bisphenol F, *IQR* interquartile range, Normalized: bisphenol concentrations were adjusted for creatinine concentrations, Imputed: undetected bisphenol concentrations were imputed to the LOQ divided by the square root of 2.

The median baseline blood pressure was 118/80 mmHg, and treatment adherence was 100%.

**Fig. 3:**
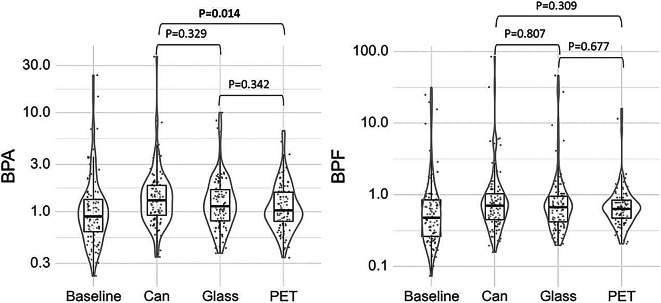
Bisphenol levels [µg/g creatinine] after Coca-Cola light consumption from different packaging among 102 women who participated in the randomized crossover intervention study. P—P value (Pairwise Tukey post hoc tests).

After the respective interventions, the geometric means of the urinary BPA concentrations were highest after consumption of Coca-Cola light from cans [1.48 µg/g creatinine, 95% CI 1.30; 1.68], followed by glass bottles [1.34 µg/g creatinine, 95% CI 1.18; 1.52] and last PET bottles [1.21 µg/g creatinine, 95% CI 1.07; 1.37] (Table 2A and Fig. 3). The BPA detection rate after Coca-Cola light consumption from cans was 49%, that from glass bottles was 35.3%, and that after PET bottles was 35.3%. We detected a significant association between the initial baseline BPA concentration and the urinary BPA concentration after the intervention, which reflects the existence of an individual profile in the BPA concentration. Each increase in baseline BPA by a factor of 10 led, on average, to a 54% increase in urinary BPA concentration after the intervention (95% CI [20%; 97%], P value 0.0008), on the original scale (back-transformed from the log-transformed model). Splitting the study population into four study phases had no appreciable impact on the outcome (P value 0.1169). Protocol adherence was assessed using the Period fixed effect in the mixed model (P value 0.3081; likelihood ratio test), which indicated no significant change in BPA concentrations across study periods, independent of other fixed effects. Visual inspection of BPA concentrations over time and across interventions further supported this, as we observed a consistent increase in BPA levels during the can packaging intervention, even when it occurred later in the study (Supplementary Material, Figs. S2 and S3). This suggest that adherence to the BPA avoidance protocol was maintained throughout the study.

**Table 2:** Mixed-effects model results for bisphenol concentration.

A Estimates for urinary BPA concentration after Coca-Cola light consumption from different packaging materials						
	BPA			BPF		
Intervention	Estimate^1^ (SE) [µg/g creatinine]	95% CI	P value^2^	Estimate^1^ (SE) [µg/g creatinine]	95% CI	P value^2^
Can	1.48 (0.09)	[1.30;1.68]	**0.018**	0.88 (0.09)	[0.71;1.08]	0.338
Glass bottle	1.34 (0.09)	[1.18;1.52]		0.83 (0.09)	[0.67;1.02]	
PET bottle	1.21 (0.08)	[1.07;1.37]		0.77 (0.08)	[0.62;0.94]	

B Comparison of different packaging materials, percentage change						
		BPA			BPF	
	Estimate^3^ (SE) [%]	95% CI	P value^4^	Estimate^3^ (SE) [%]	95% CI	P value^4^
Can vs. Glass bottles	10.7 (3.42)	[-6.43;30.92]	0.329	5.9 (4.25)	[-14.83;31.76]	0.807
Can vs. PET bottles	22.3 (3.78)	[3.37;44.64]	**0.014**	14.5 (4.59)	[-7.93;42.43]	0.309
Glass vs. PET bottles	10.5 (3.41)	[-6.60;30.68]	0.342	8.1 (4.34)	[-13.09;34.45]	0.677

^1^Regression-based predicted (geometric) marginal mean.

^2^Global test for the intervention effect (ANOVA).

^3^Percent change in (geometric) marginal mean.

^4^Pairwise Tukey post hoc tests.

*SE* standard error, *CI* confidence interval.

First and second initial urinary bisphenol concentrations in the 3^rd^ study phase did not differ significantly, and only the values obtained at the first baseline were considered in the statistical models.

The urinary BPF concentrations followed the same pattern, with the highest urinary BPF concentrations after Coca-Cola light consumption from cans, followed by glass bottles and last PET bottles [0.88 µg/g creatinine; 0.83 µg/g creatinine and 0.77 µg/g creatinine] (Table 2A and Fig. 3). Overall, the BPF concentrations were lower than the BPA concentrations.

The mean concentration of urinary BPA was significantly higher by + 22.3% (P value 0.014) after Coca-Cola light consumption from cans compared to PET bottles (Table 2B, 95% CI 3.37;44.64). Comparing cans vs. glass bottles and glass bottles vs. PET bottles, we did not find any significant differences in urinary BPA concentrations but observed a trend toward higher urinary BPA concentrations after consumption of Coca-Cola light from cans compared to glass bottles and higher urinary BPA concentrations after consumption of Coca-Cola light from glass bottles compared to PET bottles, as illustrated in Table 2B (and Supplementary Material Fig. S4). Urinary BPF concentrations did not significantly differ after the three interventions.

The blood pressure data suggested higher systolic blood pressure levels at the initial meeting and at the first meeting of the first intervention week compared to the other intervention weeks. Diastolic blood pressure levels were higher in group A at the first meeting of the first intervention week compared to the other study groups and intervention weeks. After the blood pressure measurements taken on day 1 and day 2 of the intervention week were combined, these differences were no longer evident.

A comparison of the blood pressure levels before and after the intervention overall revealed higher systolic and diastolic levels after the intervention (Table 3, also see Supplementary Material Figs. S5, S6). No differences were observed comparing the blood pressure increase between the three intervention groups (can, glass bottle, PET bottle; P value 0.755) (Table 3); hence, the increase in blood pressure after the intervention was independent of the packaging. BMI was found to be associated with blood pressure level but not with the difference in blood pressure before and after intervention (Supplementary Material Table S1). Stratifying for menopausal status did not reveal any differences of the effect of type of packaging on blood pressure between pre- and postmenopausal women (data not shown). We did not observe associations between blood pressure and menopausal status or the age of the participants (Supplementary Material Table S1).

**Table 3:** Mixed-effect models for blood pressure.

A Blood pressure analysis after Coca-Cola light consumption from different packaging materials								
Systolic					Diastolic			
Intervention	Estimate^1^ (SE) [mmHg]	95% CI	P value^2^	P value^3^	Estimate^1^ (SE) [mmHg]	95% CI	P value^2^	P value^3^
Outcome : Blood pressure difference (after-before)								
Can	1.41 (0.52)	[0.38;2.45]	**0.008**	0.755	1.03 (0.41)	[0.21;1.84]	**0.014**	0.687
Glass bottle	1.05 (0.52)	[0.02;2.08]	**0.046**		0.82 (0.41)	[0.00;1.63]	**0.050**	
PET bottle	0.95 (0.52)	[-0.08;1.98]	0.072		0.59 (0.41)	[-0.22;1.41]	0.153	
Outcome : Blood pressure after intervention								
Can	117.21 (0.56)	[116.11;118.31]		0.602	79.69 (0.49)	[78.72;80.67]		0.398
Glass bottle	116.96 (0.56)	[115.86;118.06]			80.00 (0.49)	[79.03;80.98]		
PET bottle	116.69 (0.56)	[115.59;117.79]			79.45 (0.49)	[78.47;80.42]		

B Comparison of different packaging materials, percentage change								
Systolic					Diastolic			
	Estimate^4^ (SE) [%]	95% CI	P value^5^		Estimate^4^ (SE) [%]	95% CI	P value^5^	
Outcome: Blood Pressure difference (after – before)								
Can vs. Glass bottles	0.36 (0.65)	[-1.18;1.90]	0.844		0.21 (0.50)	[-0.97;1.39]	0.907	
Can vs. PET bottles	0.46 (0.65)	[-1.07;2.00]	0.756		0.43 (0.50)	[-0.75;1.62]	0.662	
Glass vs. PET bottles	0.10 (0.65)	[-1.43;1.64]	0.986		0.22 (0.50)	[-0.96;1.41]	0.896	
Outcome: Blood pressure after intervention								
Can vs. Glass bottles	0.25 (0.52)	[-0.96;1.47]	0.875		-0.31 (0.41)	[-1.28;0.66]	0.730	
Can vs. PET bottles	0.52 (0.52)	[-0.70;1.74]	0.573		0.25 (0.41)	[-0.72;1.21]	0.821	
Glass vs. PET bottles	0.27 (0.52)	[-0.95;1.48]	0.864		0.56 (0.41)	[-0.41;1.52]	0.366	

^1^Predicted marginal means.

^2^Two-sided significance test from zero (performed with function ‘emmeans’ from package ‘emmeans’).

^3^Global test for the intervention effect (ANOVA).

^4^Marginal mean differences.

^5^Pairwise Tukey post hoc tests.

*SE* standard error, *CI* confidence interval.

## Discussion

In this randomized crossover intervention trial, we aimed to compare urinary concentrations of BPA and its analogues after soda consumption from cans, PET bottles and glass bottles. Additionally, we evaluated whether blood pressure was affected by soda consumption from cans, PET bottles and glass bottles and whether blood pressure changes are dependent on the packaging material.

We detected significantly higher BPA concentrations in the urine samples of our study participants after soda consumption from cans compared to soda consumption from PET bottles.

This finding is consistent with several previous studies that reported higher urinary BPA concentrations after beverage and food consumption from cans compared to other packaging materials. In 2011, we were the first to detect an increase in urinary BPA concentrations after canned food (soup) consumption compared to freshly prepared food (soup) consumption in a crossover intervention study conducted in the U.S. The increase in specific gravity (SG)-adjusted urinary BPA concentration was 1221%^30^. Consequently, Bea and Hong examined urinary BPA concentrations after beverage consumption from cans. In their intervention study conducted in South Korea, 60 elderly study participants were assigned to consume soy milk from only cans, cans and glass bottles or only glass bottles, and > 1600% higher urinary BPA levels were observed after canned soy milk consumption compared to consumption of soy milk from glass bottles. Their urinary BPA concentrations were considerably higher (20.65 ± 8.61 µg/g creatinine) than those we found in our study participants (1.48 µg/g creatinine)^9^; however, South Korea is a country with high BPA exposure^49^. Two other studies examining the influence of BPA-containing beverage containers (here made of polycarbonate) in the USA and China reported urinary BPA concentrations of 2.0 μg/g creatinine and 2.75 μg/g creatinine after beverage consumption, respectively^50,51^. Intervention studies involving canned food, from Taiwan and the US, found similar urinary BPA concentrations after canned food consumption to those reported by Bae and Hong (20.4 μg/g creatinine)^30,52^. The very high concentrations observed after the soy milk intervention could either be due to higher amounts of BPA used in the lining of beverage cans in South Korea or to differences in the composition of soy milk compared to water-based beverages. Furthermore, we detected a difference in the urinary BPA concentration only after canned soda consumption in comparison to PET bottles and not to glass bottles. While these differing results remain unclear, our sample size was limited, and bisphenol levels were generally modest. However, in light of these differing results, the elevated BPA levels observed after soda consumption from cans in comparison with soda consumption from PET bottles have to be interpreted cautiously.

The increase in urinary BPA concentrations in our study from 2011 (> 1000%) after canned soup consumption compared to fresh soup consumption is similar to the rise in urinary BPA concentrations reported by Bea and Hong (> 1600%) after canned soy milk consumption compared to soy milk from glass bottles. In comparison to the change of urinary BPA concentrations observed in the present study (22.3%; can vs. PET bottle), these values are substantially higher. Manufacturers may have voluntarily reduced the BPA content in the epoxy resin of cans more recently; however, the chemicals used in the epoxy resin lining is proprietary information. Additionally, epoxy resin linings differ in constitution and thickness^53,54^.

Bisphenol detection rates in our study were lower than those reported in most biomonitoring studies. A recent investigation in Germany reported BPA levels in 96% of 515 randomly chosen subsamples of the KiGGS Wave 2 study of 3- to 17-year-old children and adolescents. The mean urinary BPA concentration in their urine samples collected between 2014 and 2017 was 1.67 µg/g creatinine^55^. They also compared their data to values from 2003 to 2006 and reported a 26% lower mean bisphenol concentration, suggesting a decline in BPA exposure over time. A systematic review and meta-analysis summarized adult BPA exposure in different populations from 15 independent studies with a total sample size of 28,353 participants and detected exposure in more than 90% of the study population. Nine of the studies reported median urinary BPA concentrations adjusted for creatinine concentrations, with medians ranging from 1.20 μg/g creatinine to 2.41 μg/g creatinine^49^. Pooled BPA concentrations exhibit marginally higher levels for men than for women. One explanation for the generally lower BPA exposure rates and BPA levels detected in our study population may be that individuals volunteering to participate in an intervention study are more health conscious, have an awareness of EDCs and their sources, and purposefully try to avoid exposure to EDCs. Moreover, we asked our participants to avoid sources of BPA, such as consuming food or drink from cans and touching receipt paper, during the wash-out and intervention periods.

In addition to BPA, we detected BPF, BPS and BPE in some of the urine samples but to a lesser extent and independent of beverage packaging. Data on exposure to BPA alternatives are rather limited. One study with 616 urinary samples from the United States (U.S.) collected in different years from 2000 to 2014 found highest detection rates for BPA, followed by BPF and BPS. This pattern is comparable to that observed in our study. BPA exposure has decreased over the years, whereas BPF exposure has increased^39,56^.

Another finding of our study is that systolic and diastolic blood pressure increased after the interventions in general, but the increase in blood pressure was independent of whether the soda was consumed from cans, PET bottles, or glass bottles. As there was no difference between the intervention groups, the most likely explanation may be the caffeine content of the Coca-Cola light, as caffeine consumption may be associated with an acute rise in blood pressure^57^. Our observations contrast with those of the intervention study of Bae and Hong, who reported higher systolic blood pressure levels after the consumption of canned soy milk compared to glass bottled soy milk; however, our increase in BPA concentrations after soda consumption from cans was considerably less pronounced than that reported in the South Korean study. Moreover, their study included elderly participants, and 27 of 60 participants were already hypertensive. After the hypertensive participants were excluded from their analysis, the difference in blood pressure before and after the intervention was no longer significant^9^.

An analysis of NHANES data collected from 2003 to 2016 with samples from 9243 participants found, that BPA exposure enhances cardiovascular mortality risk especially for women^58^. The study population in the study of Bae and Hong consisted of 93.3% female participants, too. Estrogen is known to have a protective effect on the cardiovascular system and pre-menopausal women have a lower incidence of coronary artery disease than post-menopausal women^59^. We chose female participants to be able to examine the association of pre- and postmenopausal hormone status with blood pressure change before and after the intervention. As BPA is thought to disrupt normal cell function by signaling estrogen activity and regulating gene expression but simultaneously competing with estrogen for the binding of membrane estrogen receptors and therefore blocking estrogen activity as well as acting as an androgen antagonist, it is possible that a modulation in blood pressure by BPA can be mediated by a combined interaction effect of BPA and endogenous estradiol on the estrogen receptors in the blood vessels^4–7^. We did not observe any differences in the effect of the different interventions on blood pressure between premenopausal and postmenopausal women. However, the effect of the interventions on the participants’ blood pressure in our study is most likely independent of BPA, which makes this result plausible.

A limitation of our study is the collection of spot urine samples, which can vary by urine volume, concentration, and other factors. To address this concern, we pooled two samples collected following the same intervention to reduce intra-individual variability. Urinary BPA concentrations were adjusted for creatinine to account for variability in urine dilution. Another limitation is that we cannot ensure that the changes in bisphenol concentrations were only caused by the intervention, however, exposure to other sources of bisphenol would have been random, especially since the intervention was blinded to the participants. Random variation due to BPA exposure from other sources may have occurred, however, it is unlikely that this would have deterred any meaningful systematic BPA variation between the three intervention periods. Also, these concerns can be mitigated by our previous findings, in which, following a similar study design and using a similar sample size, we found polycarbonate bottle use to significantly elevate urinary BPA concentrations, and canned soup consumption to significantly elevate urinary BPA concentrations, respectively^30,50^. We were not able to measure urinary bisphenol concentrations after each wash-out period which might have provided additional information on baseline levels prior to each intervention. We designed the wash-out period to be substantially longer than the time until complete elimination of bisphenols from the organism to exclude the presence of any carry-over effect. In order to avoid batch effects we did not analyse urine samples right after collection but collected all samples and analysed them at the same time. Thus, some of the urine samples in this study were stored for 17 months before analysis, but prior studies indicate, that conjugated bisphenol concentrations remain stable at subfreezing concentrations during this time period^56^. Moreover, our study population only included generally healthy women, hence we cannot generalize our findings to men, children, or individuals with pre-existing disorders.

To the best of our knowledge, this is the first intervention trial comparing urinary bisphenol levels after soda consumption from cans, PET bottles and glass bottles. The randomized crossover design is a strength of our study and enables us to directly compare the three interventions without interindividual differences and to eliminate any effects of the sequence in which the interventions were given. The collection of two urine samples per intervention reduced intraindividual fluctuation.

With the increasing substitution of other bisphenols for BPA, it will be important to learn more about the use of BPA alternatives and their health effects. As bisphenols are not the only class of chemicals with human health concerns and humans are exposed to a mixture of chemicals, it seems sensible to work on strategies to completely replace substances that pose a known health risk.

In conclusion, our study suggests that canned soda may still increase the amount of BPA excreted in the urine of humans in spite of substitution measures in recent years. Given the potential of bisphenol exposure from canned food and beverage consumption, as well as the availability of alternatives in can linings, the complete removal of BPA from cans is an attractive option for mitigating human exposure to bisphenols.

## Supplementary Information

Supplementary Information.

## Data Availability

The datasets used and/or analysed during the current study are available from the corresponding author on reasonable request.

## Abbreviations

ACN: Acetonitrile
AEI: Advanced electron ionization
ANOVA: Analysis of variance
BMI: Body mass index
BPA: Bisphenol A
BPE: Bisphenol E
BPF: Bisphenol F
BPS: Bisphenol S
BSTFA: N,O-bis-(trimethylsilyl)-trifluoracetamide
CI: Confidence interval
DRKS: Deutsches register Klinische Studien
ECHA: European chemical agency
EDC: Endocrine-disrupting chemical
EFSA: European food safety authority
GC-AEI-MS/MS: Gas chromatography-advanced electron ionization-tandem mass spectrometry
GC–MS/MS: Gas chromatogram-tandem mass spectrometry
ICC: Intraclass correlation coefficient
IQR: Interquartile range
LOQ: Limit of quantification
MeOH: Methanol
MtBSTFA: N-tert-Butyldimethylsilyl-N-methyltrifluoracetamid
NaAc: Sodium acetate
PET: Polyethylene terephthalate
SE: Standard error
SG: Specific gravity
SPE: Solid-phase extraction
TBBA: Tetrabromobisphenol A
TBDMSCl: Tert-butyldimethylchlorsilan
US: United States
ΔBP: Change in blood pressure
ΔDBP: Change in diastolic blood pressure
ΔSBP: Change in systolic blood pressure
