# Supplementary material for: Urinary bisphenol levels and blood pressure after soda consumption from cans, PET and glass bottles
Source: Sci Rep. 2025 Sep 12;15:32477. doi: 10.1038/s41598-025-18459-z (PMC12432166; doi:10.1038/s41598-025-18459-z)
Supplement: Supplementary file 1 — Supplementary Information. [file 41598_2025_18459_MOESM1_ESM.docx]

**Urinary bisphenol levels and blood pressure after soda consumption from cans, PET and glass bottles.**

Leonie Plachetka^1^

Virginie Stanislas^1^

Alexander Bauer^1^

Thomas Göen^2^

Heike Denghel^2^

Karin B. Michels^1,*^

^1^ Institute for Prevention and Cancer Epidemiology, Faculty of Medicine and Medical Center, University of Freiburg, 79110 Freiburg, Germany

^2^ Institute and Outpatient Clinic of Occupational, Social and Environmental Medicine, Friedrich-Alexander-Universität Erlangen-Nürnberg, 91054 Erlangen, Germany

Contact information for the corresponding author:

Name: Karin B. Michels, ScD, PhD

e-mail: tumorepidemiologie@uniklinik-freiburg.de

postal address: Institute for Prevention and Cancer Epidemiology, Elsaesserstr. 2, 79110 Freiburg, Germany

Conflict of interest declaration

The authors declare that they have nothing to disclose.

Funding

This project was funded by the Deutsche Forschungsgemeinschaft (DFG, German Research Foundation) under project number MI 1461/8-1 awarded to KBM.

Universal Trial Number/ DRKS-ID

U1111-1244-7033/ DRKS00019922 (Registration Date 29/11/2019)

**Supplementary Material**


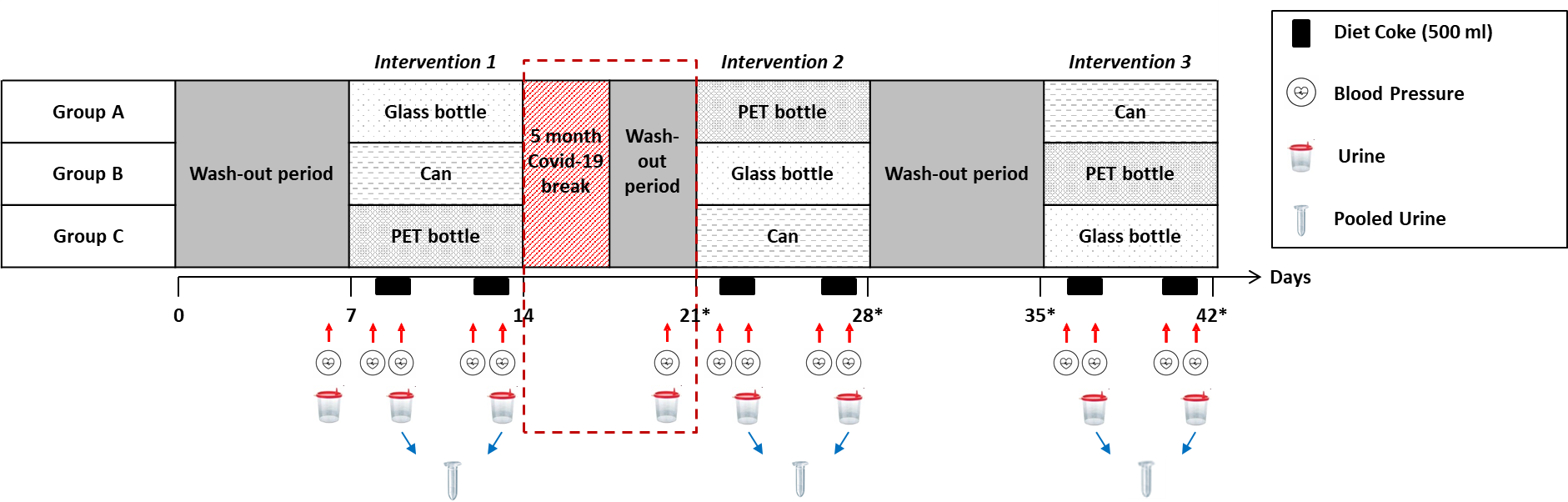


**Figure S1:** Study design of study phase III (with Covid-19 break) of the randomized, single-blind, crossover intervention trial**.**

*The study participants underwent the same study protocol as presented and explained in Figure 2 of the main manuscript except a study break of 5 months due to the Covid-19 pandemic. After the break the study was proceeded with a 1-week wash-out period and an additional baseline urine sample collection and baseline blood pressure measurement.*

*PET - Polyethylene terephthalate*

** Regular study days PLUS duration of the Covid-19 break*


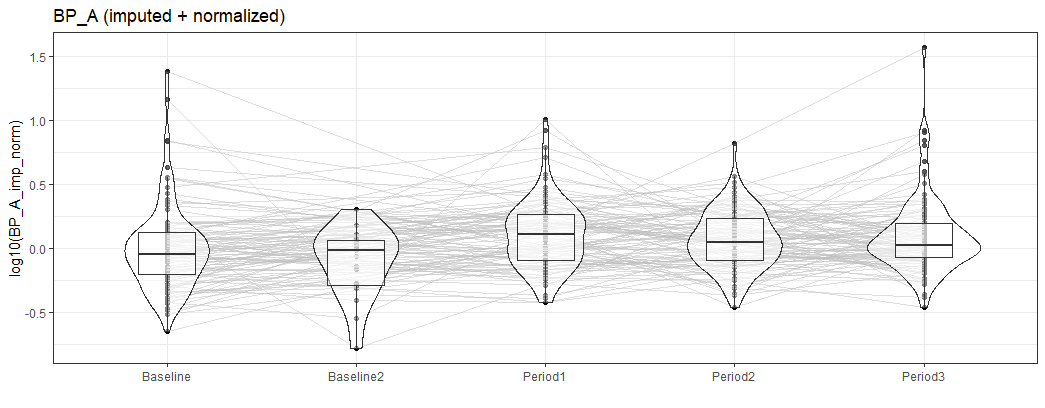


**Figure S2:** BPA concentration over study periods.


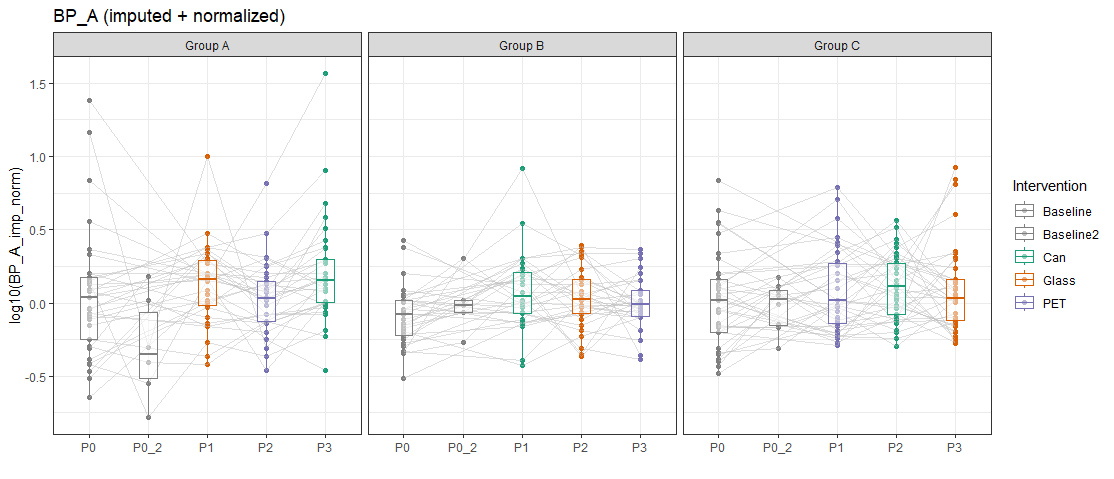


**Figure S3:** BPA concentration over study periods stratified by study groups and interventions.

*PET - Polyethylene terephthalate*

*P0 - Baseline*

*P0_2 - Second Baseline*

*P1 - Intervention Week 1*

*P2 - Intervention Week 2*

*P3 - Intervention Week 3*


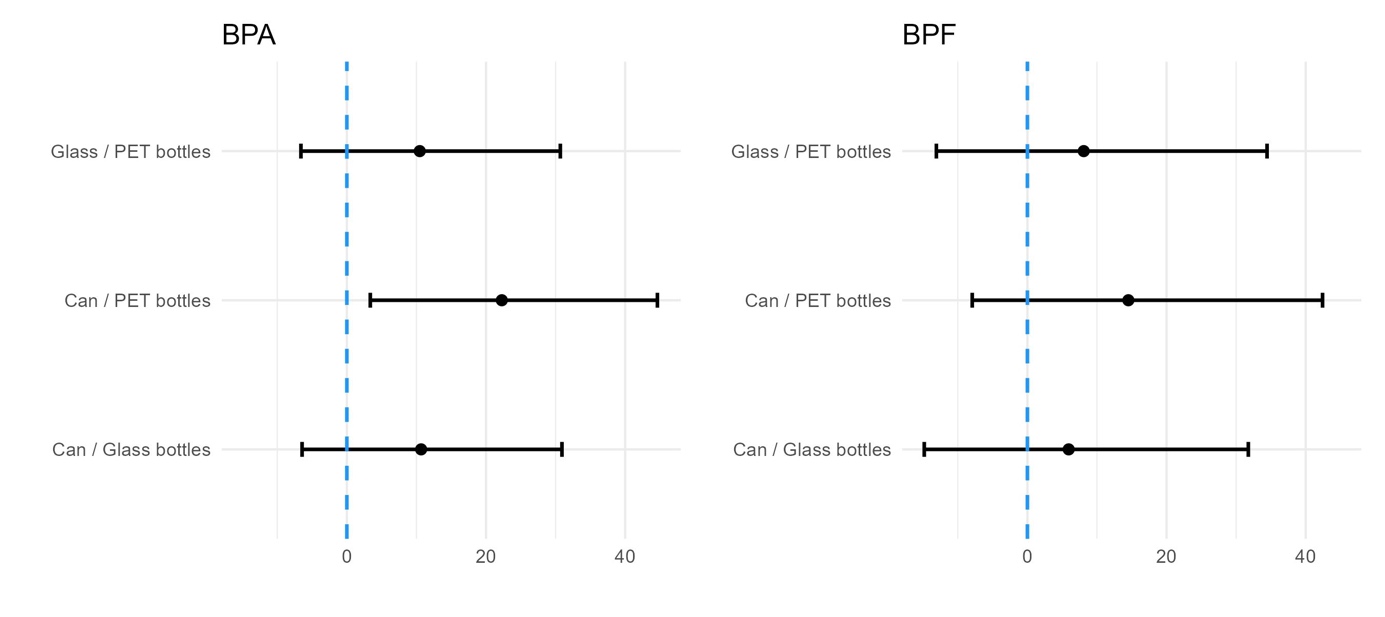


**Figure S4:** Percent change and confidence intervals of the comparison of urinary BPA and BPF concentrations after comparing glass vs. PET bottles, can vs. PET bottles and can vs. glass bottles.


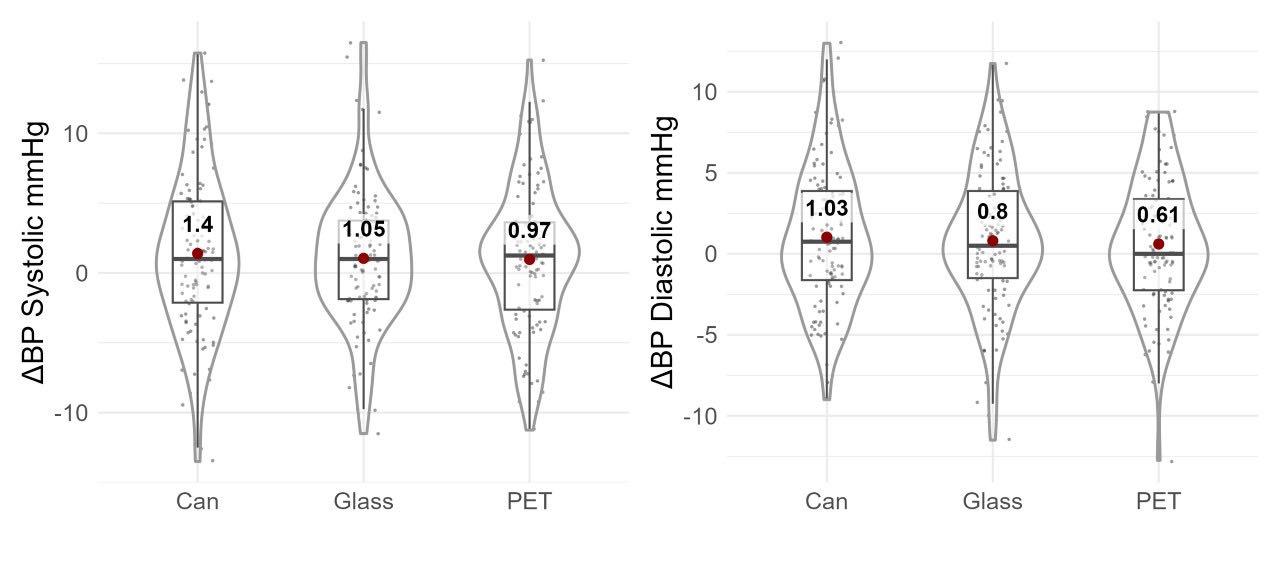


**Figure S5:** *Systolic and diastolic blood pressure differences (ΔBP = after intervention – before intervention; mmHg) following Coca-Cola light* *consumption from can, glass bottle and PET bottle.*

*Red dot - Observed mean values [mmHg] in each category*


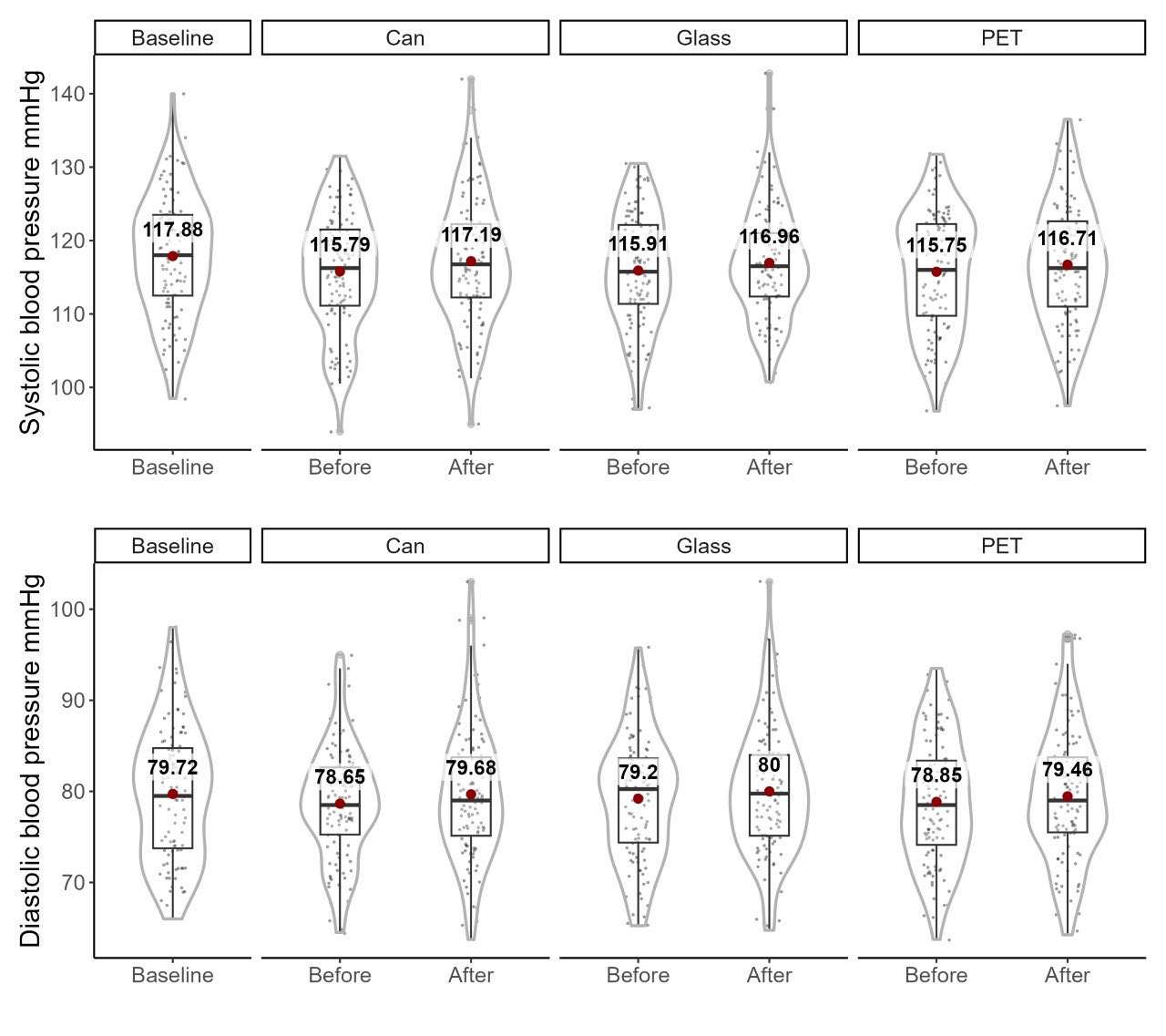


**Figure S6:** *Systolic and diastolic* *blood pressure levels [mmHg] at baseline, and before and*

*after Coca-Cola light consumption from can, glass bottle and PET bottle.*

*Red dot - Observed mean values [mmHg] in each category*

**Table S1:** Explorative analysis of effects of age, BMI and menopausal status on systolic and diastolic blood pressure.

|  | **Systolic** | | | **Diastolic** | | | |
| --- | --- | --- | --- | --- | --- | --- | --- |
| **Covariate** | **Estimate**^1^  **[mmHg]** | **95% CI** | **P value** | **Estimate**^1^  **[mmHg]** | **95% CI** | **P value** |  |
| *Outcome : Blood pressure difference (after-before)* | | | | | | |  |
| Age | -0.06 | [-0.13;0,02] | 0.1292 | -0.03 | [-0.09;0.03] | 0.3927 |  |
| BMI | 0.11 | [-0.12;0.33] | 0.3518 | 0.06 | [-0.13;0.24] | 0.5324 |  |
| Menopause | -1.41 | [-2.95;0.12] | 0.0745 | -1.08 | [-2.36;0.19] | 0.0984 |  |
| *Outcome : Blood pressure after intervention* | | | | | | |  |
| Age | 0.06 | [-0.07;0.19] | 0.3510 | 0.08 | [-0.04;0.20] | 0.1951 |  |
| BMI | 0.55 | [0.24;0.87] | 0.0008 | 0.35 | [0.07;0.63] | 0.0174 |  |
| Menopause | -0.50 | [-3.27;2.26] | 0.7209 | 1.35 | [-1.24;3.94] | 0.3088 |  |

*The effects of all three parameters were estimated by individually adding each parameter to each respective final mixed regression model. For each parameter, we report the estimate, corresponding 95% confidence interval (95% CI) and P value. For the outcome: blood pressure after intervention, BMI was part of the final linear mixed regression model.*

*^1^Mixed regression model-based effect estimates*

**Table S2:** Drop out characteristics

| Study phase | Number of dropouts | Age median | Age mean (std. deviation) |
| --- | --- | --- | --- |
| Overall | 18 | 53 | 50.2 (10.5) |
| 1 | 6 | 52 | 52.7 (6.3) |
| 2 | 3 | 33 | 39.0 (12.2) |
| 3 | 9 | 55 | 52.2 (10.7) |
| 4 | 0 | - | - |

*Supplementary information on study participants and drop outs*

Overall, we included 122 women in the study, 19 of whom did not complete the study (Table S2). The reasons for dropout were lack of time, pregnancy, illness, and changes in everyday life due to COVID-19. One participant had missing urine samples for two of the interventions and was removed from the bisphenol analysis.

*Supplementary information on power calculations*

A sample size of 100 study participants was determined by power calculations based on previous studies. We expected to observe urinary levels of approximately 10-15 ug/L after canned soda consumption compared to 2-3 ug/L after bottled (glass or PET) soda consumption. We anticipated >95% power to detect a 8 ug/L difference in BPA concentration with 100 individuals, assuming a standard deviation of 1-2 ug/L for the difference in the BPA levels after canned soda vs. bottled soda.

A study comparing canned vs. bottled soy milk found a ΔSBP of 4.5 mmHg.^9^ We anticipated >90% power to detect a ΔBP of 4 mmHg with a sample size of 100 individuals.

*Supplementary information on the measurement of additional bisphenols below the limit of quantification*

Standards for Bisphenol AF (BPAF, 2,2-bis-(4-hydroxyphenyl)hexafluorpropane, purity 99%), Bisphenol AP (BPAP, 1,1-bis-(4-hydroxyphenyl)-1-phenylethane, purity 99%), Bisphenol B (BPB, 2,2-bis-(4-hydroxyphenyl)butane, purity 98%), Bisphenol BP (BPBP, bis-(4-hydroxyphenyl)-diphenylmethane, purity 98%), Bisphenol C (BPC, 2,2-bis-(4-hydroxy-3-methylphenyl)propane, purity 99%), Bisphenol E (BPE, 1,1-bis-(4-hydroxyphenyl)ethane, purity 98%), and Bisphenol P (BPP, 1,4-Bis-(2-(4-hydroxyphenyl)-2-propyl)benzol, purity 99%) and Bisphenol S (BPS, bis-(4-hydroxyphenyl)sulfone, purity 98%) were purchased from Sigma-Aldrich (Steinheim, Germany).

Matched isotope-labeled standards of Bisphenol AF - ^13^C_12_ (BPAF - ^13^C_12_, chemical purity >98%, isotopic purity 99%), Bisphenol B - ^13^C_12_ (BPB - ^13^C_12_, chemical purity >98%, isotopic purity 99%) and Bisphenol S - ^13^C_12_ (BPS - ^13^C_12_, chemical purity >98%, isotopic purity 98%) were obtained from Cambridge Isotope Laboratories (Andova, USA). Bisphenol AP - ^13^C_6_ (BPAP - ^13^C_6_, chemical purity 97%, isotopic purity 98.6%), Bisphenol P - ^13^C_4_ (BPP - ^13^C_4_, chemical purity 99.29%, isotopic purity 98.9%) and Bisphenol Z - ^13^C_12_ (BPZ - ^13^C_12_, chemical purity 95%, isotopic purity 98.8%) were purchased from Biozol (Eching, Germany).
